# Supplementary material for: Enhanced Proliferative and Osteogenic Potential of Periodontal Ligament Stromal Cells
Source: Biomedicines. 2023 May 3;11(5):1352. doi: 10.3390/biomedicines11051352 (PMC10216378; doi:10.3390/biomedicines11051352)
Supplement: Supplementary file 1 [file biomedicines-11-01352-s001.zip › biomedicines-2300680-supplementary.pdf]

Supplementary information

Enhanced Proliferative and Osteogenic Potential of Periodontal Ligament Stromal Cells

Laura Alves [1,2], Vanessa Machado [3,4], João Botelho [3,4], José João Mendes [3,4], Joaquim M.S. Cabral [1,2], Cláudia L. da Silva [1,2] and Marta S. Carvalho [1,2,*]

[1] Department of Bioengineering and iBB, Institute for Bioengineering and Biosciences, Instituto Superior Técnico, Universidade de Lisboa, Av. Rovisco Pais, 1049-001 Lisboa, Portugal

[2] Associate Laboratory i4HB – Institute for Health and Bioeconomy at Instituto Superior Técnico, Universidade de Lisboa, Av. Rovisco Pais, 1049-001 Lisboa, Portugal

[3] Clinical Research Unit, Egas Moniz Center for Interdisciplinary Research, Egas Moniz School of Health and Science, Almada 2829-511, Portugal.

[4] Evidence-Based Hub, Egas Moniz Center for Interdisciplinary Research, Egas Moniz School of Health and Science, Almada 2829-511, Portugal.

* Correspondence: martacarvalho@tecnico.ulisboa.pt

Supplementary Figure S1

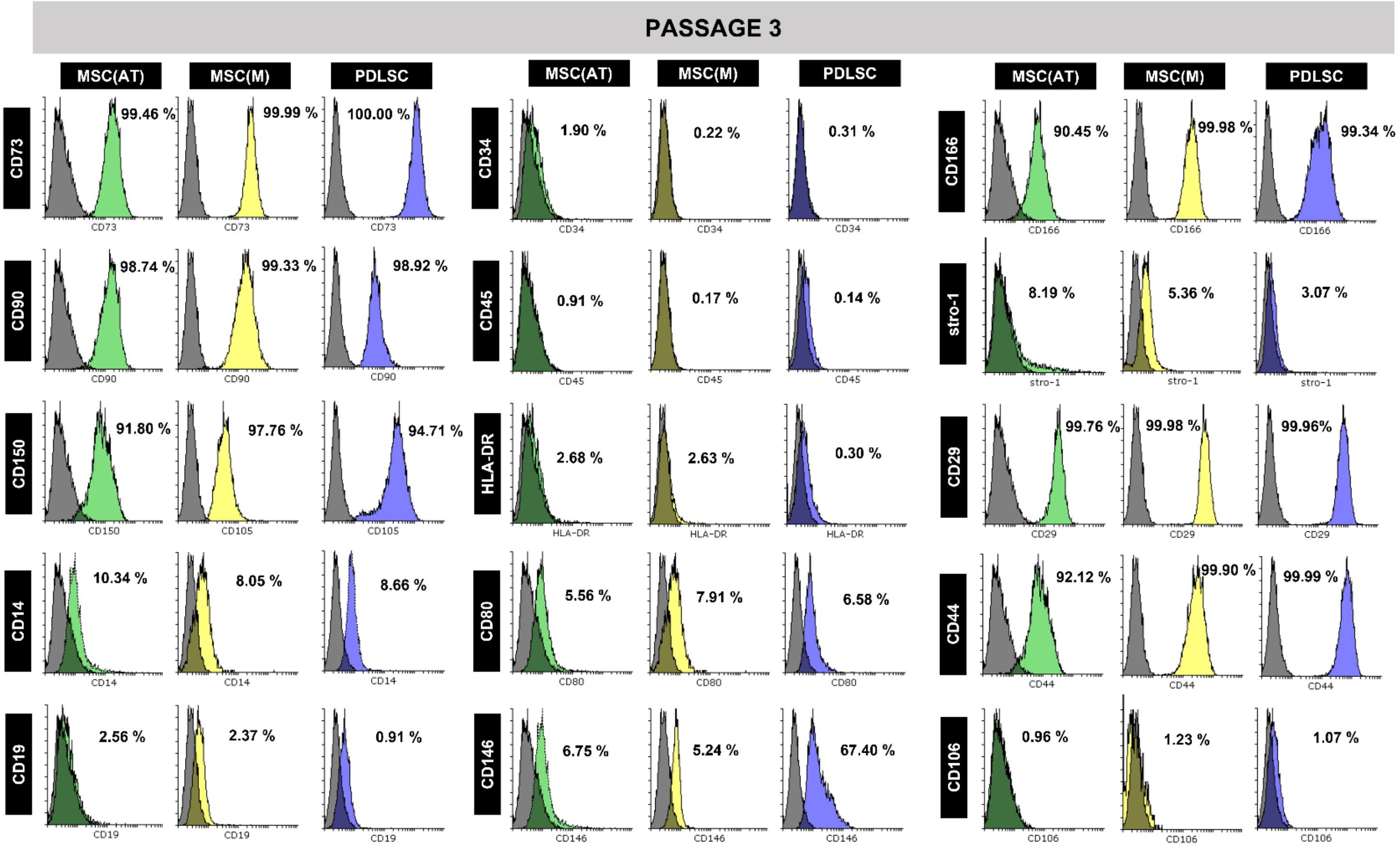

Supplementary Figure S1. Flow cytometry analysis of MSC(AT), MSC(M) and PDLSC at passage 3. Grey histograms are the controls for each cell source.

Supplementary Figure S2

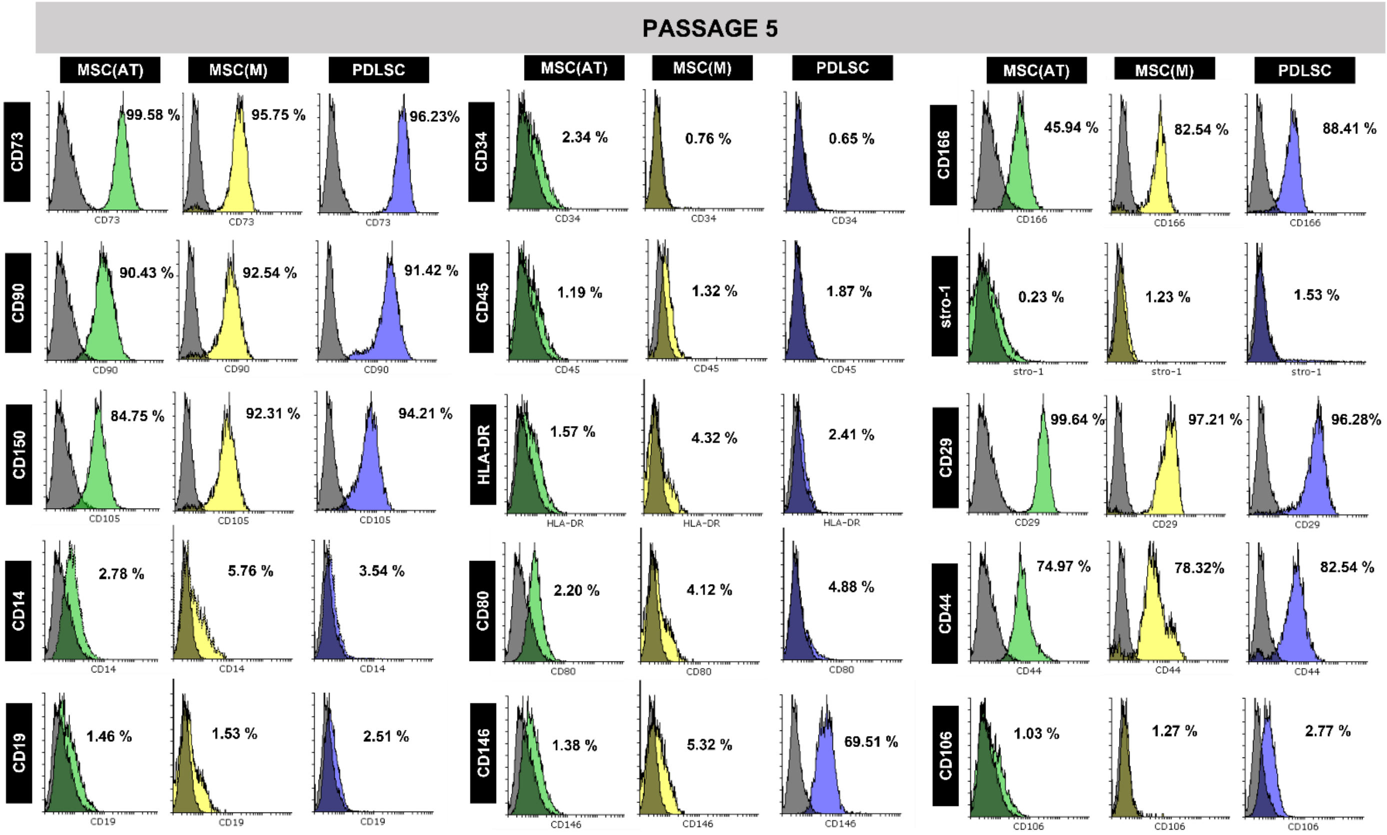

Supplementary Figure S2. Flow cytometry analysis of MSC(AT), MSC(M) and PDLSC at passage 5. Grey histograms are the controls for each cell source.

Supplementary Figure S3

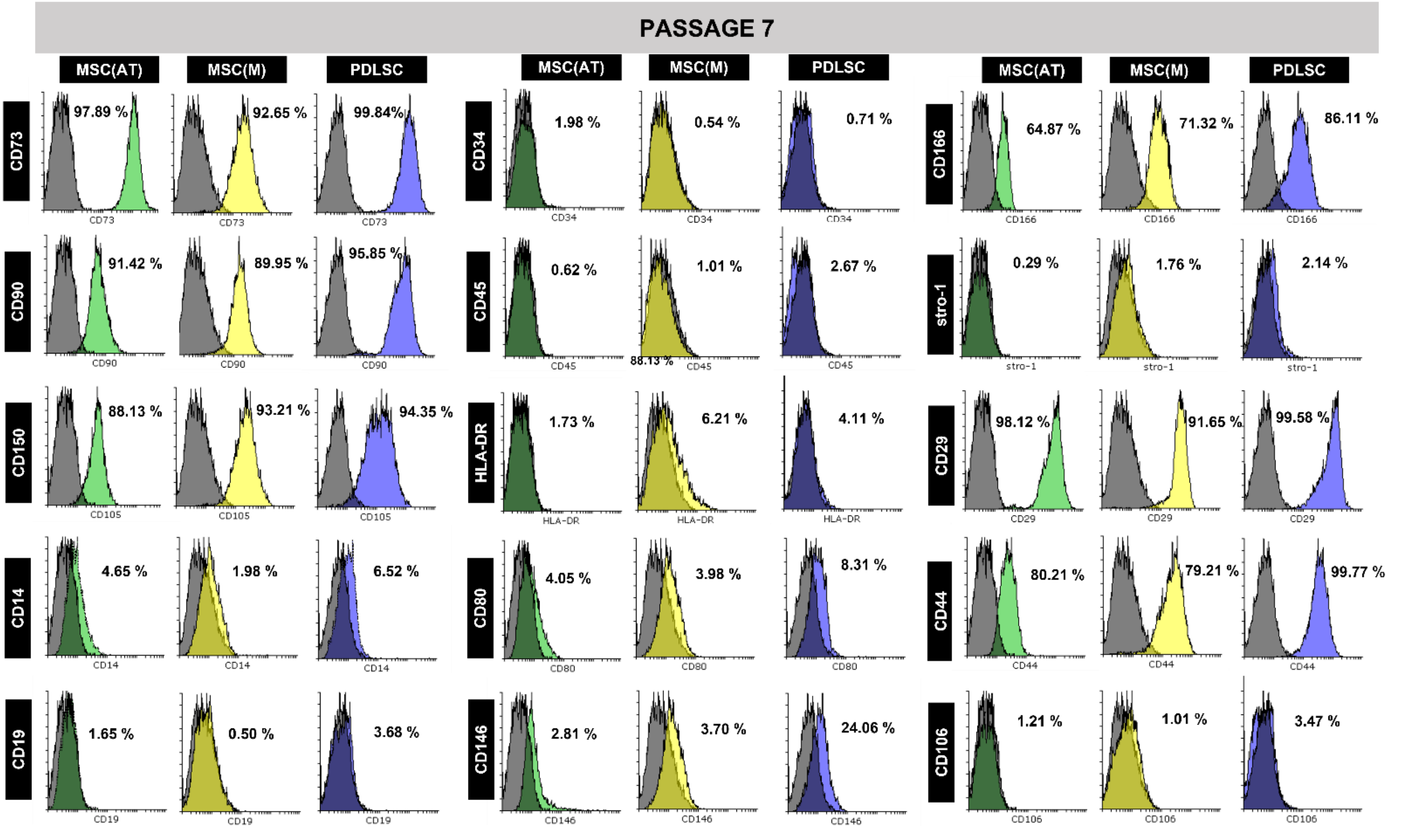

Supplementary Figure S3. Flow cytometry analysis of MSC(AT), MSC(M) and PDLSC at passage 7. Grey histograms are the controls for each cell source.

Supplementary Table S1

Supplementary Table S1. Exponential regression parameters and respective coefficient of determination (R^2) estimated using MS Excel for periodontal ligament stromal cells (PDLSC), bone marrow (MSC(M)) and adipose tissue-derived mesenchymal stromal cells (MSC(AT)) cultured for 9 days in expansion conditions and seeded at 1500 cells/cm^2 and 3000 cells/cm^2 in 12 well-plates (4 cm^2). N_0 corresponds to initial number of seeded cells and μ is the cell growth rate.

	**N_0 (cells)**	**μ ($days^{-1}$)**	**R^2**
**PDLSC 1500 cells/cm^2**	6000	0.531	0.925
**PDLSC 3000 cells/cm^2**	12000	0.496	0.916
**MSC(M) 1500 cells/cm^2**	6000	0.176	0.900
**MSC(M) 3000 cells/cm^2**	12000	0.165	0.880
**MSC(AT) 1500 cells/cm^2**	6000	0.339	0.913
**MSC(AT) 3000 cells/cm^2**	12000	0.220	0.937
